# Licochalcone A Protects the Blood–Milk Barrier Integrity and Relieves the Inflammatory Response in LPS-Induced Mastitis

**DOI:** 10.3389/fimmu.2019.00287

**Published:** 2019-02-25

**Authors:** Wenjin Guo, Bingrun Liu, Yunhou Yin, Xingchi Kan, Qian Gong, Yanwei Li, Yu Cao, Jianfa Wang, Dianwen Xu, He Ma, Shoupeng Fu, Juxiong Liu

**Affiliations:** ^1^College of Animal Science and Veterinary Medicine, Jilin University, Changchun, China; ^2^Laboratory of Biomolecular Research, Division of Biology and Chemistry, Paul Scherrer Institute, Villigen, Switzerland; ^3^School of Communication, Guizhou Minzu University, Guiyang, China; ^4^College of Animal Science and Veterinary Medicine, Heilongjiang Bayi Agricultural University, Daqing, China

**Keywords:** mastitis, blood–milk barrier, AKT/NF-κB, MAPK, mMECs, licochalcone A

## Abstract

**Background/Aims:** Mastitis is an acute clinical inflammatory response. The occurrence and development of mastitis seriously disturb women's physical and mental health. Licochalcone A, a phenolic compound in *Glycyrrhiza uralensis*, has anti-inflammatory properties. Here, we examined the effect of licochalcone A on blood-milk barrier and inflammatory response in LPS-induced mice mastitis.

**Methods:** *In vivo*, we firstly established mice models of mastitis by canal injection of LPS to mammary gland, and then detected the effect of licochalcone A on pathological indexes, inflammatory responses and blood-milk barrier in this model. *In vivo*, Mouse mammary epithelial cells (mMECs) were treated with licochalcone A prior to the incubation of LPS, and then the inflammatory responses, tight junction which is the basic structure of blood-milk barrier were analyzed. Last, we elucidated the anti-inflammatory mechanism by examining the activation of mitogen-activated protein kinase **(**MAPK) and AKT/NF-κB signaling pathways *in vivo* and *in vitro*.

**Result:** The *in vivo* results showed that licochalcone A significantly decreased the histopathological impairment and the inflammatory responses, and improved integrity of blood-milk barrier. The *in vitro* results demonstrated that licochalcone A inhibited LPS-induced inflammatory responses and increase the protein levels of ZO-1, occludin, and claudin3 in mMECs. The *in vivo* and *in vitro* mechanistic study found that the anti-inflammatory effect of licochalcone A in LPS-induced mice mastitis was mediated by MAPK and AKT/NF-κB signaling pathways.

**Conclusions and Implications:** Our experiments collectively indicate that licochalcone A protected against LPS-induced mice mastitis via improving the blood–milk barrier integrity and inhibits the inflammatory response by MAPK and AKT/NF-κB signaling pathways.

## Introduction

Mastitis, a common clinical disease in both animals and humans (1, 2), is an acute condition with obvious symptoms of inflammation. Uncontrolled mastitis can be extremely harmful to lactating women (3, 4). Moreover, mastitis has a potential negative effect on breastfeeding and is a main cause of considerable physical and psychological pain during lactation (5, 6). In general, antibiotic treatment is the most commonly used strategy for treating acute mastitis. However, while antibiotics generally have a positive effect on bacteriostasis (7), they are not useful for controlling the development of subsequent inflammation (8).

**Figure 1:**
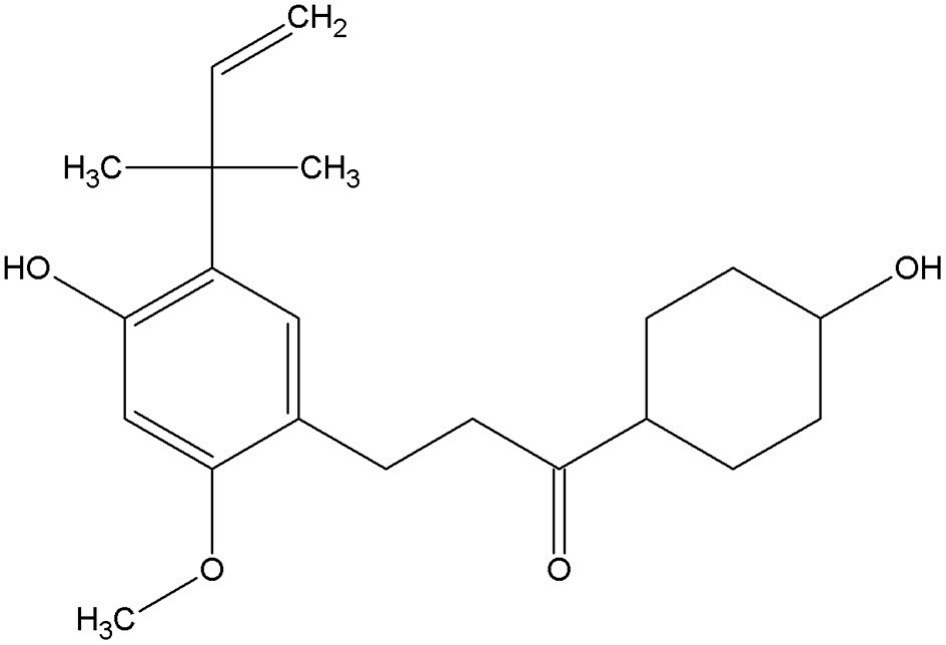
Structure of licochalcone A.

Licochalcone A is a phenolic chalcone compound initially isolated from *Glycyrrhiza uralensis* in 1975 by Saitoh (9). The structure of licochalcone A and various biological activities have been established to date (Figure 1) (10), including anti-inflammatory (11), anti-oxidation (12), antiseptic, and anti-tumor properties (13, 14). As a traditional Chinese medicine monomer with anti-inflammatory and anti-bacterial properties (15, 16), it has several advantages over traditional antibiotics. Drug resistance to this compound is relatively uncommon (17), along with a significant inhibitory effect on inflammation. Therefore, we speculate that licochalcone A may also have a protective effect on mastitis.

The blood–milk barrier is important for mammary gland resistance to the external environment (18). Destruction of the blood–milk barrier aggravates bacterial infection and promotes the development of inflammation (19). Acute mastitis is usually caused by infection of gram-negative bacteria and explosive proliferation (20). *Escherichia coli*, a common gram-negative bacterium, is widely present in the external environment and an important cause of acute mastitis (21). Lipopolysaccharide (LPS) in *E. coli* can cause severe immune response to mammary gland (22). However, LPS also destroys the blood–milk barrier and promotes the occurrence and development of inflammation (23). So in our experiments, mouse mastitis model was constructed using LPS extracted from *E. coli*. LPS can activate MAPK and AKT/NF-κB signaling pathways, which causes mouse mammary epithelial cells (mMECs) to release a large number of pro-inflammatory factors, destroy the epithelial barrier and accelerate the development of mastitis (24, 25). Therefore, inhibiting the release of pro-inflammatory factors in mMECs and protecting the tight junction of mMECs may play an important role in alleviating mastitis. So the purpose of our study was to explore the effect of licochalcone A on inflammatory responses and blood-milk barrier in LPS-induced *in vivo* and *in vitro* mastitis models.

## Materials and Methods

### Drugs and Reagents

Licochalcone A (purity >98%) was obtained from Chengdu Pufei De Biotech Co., Ltd, dimethylsulfoxide (DMSO) from Sigma Chemical Co. (St. Louis, MO, USA), and fetal bovine serum (FBS) from Clark Bioscience Co. (Richmond, va, USA). Dulbecco's modified Eagle's medium (DMEM) for cell culture was obtained from Invitrogen-Gibco (Grand Island, NY, USA). Cell Counting Kit-8 (CCK8) was acquired from Saint-Bio Co. (Shanghai, China). 3,3′,5,5′-Tetramethylbenzidine, Resorcinol, H_2_O_2_ and Hepes were purchased from Sigma Chemical Co. (St. Louis, MO, USA). Enzyme-linked immunosorbent assay (ELISA) kits for TNF-α, IL-6 and IL-1β were obtained from Biolegend Co. (San Diego, CA, USA). The primary antibodies AKT, p-AKT, ERK1/2, p-ERK1/2, JNK1/2, p-JNK1/2, P38, p-P38, IκBα, p-IκBα, NF-κB-p65, and p-NF-κB-p-p65 were purchased from Cell Signaling Technology (CST) (Danvers, MA, USA). ZO-1 was acquired from Proteintech (Chicago, IL, USA). Claudin-3, COX-2 and iNOS were purchased from Abcam (Cambrige, UK), occludin from Thermo Fisher (Waltham, MA, USA) and β-tubulin from Bosterbio (CA, USA). The secondary antibodies used in this study, including Alexa Fluor 594 or Alexa Fluor 488 conjugate donkey anti-rabbit or anti-mouse IgG (H+L) highly cross-adsorbed secondary antibody were purchased from Life Technologies (Carlsbad, CA, USA) and HRP-conjugated goat anti-mouse and goat anti-rabbit secondary antibodies were purchased from Bosterbio.

### Cell Culture

mMECs purchased from American Type Culture Collection (ATCC® CRL-3063™) were cultured in DMEM containing 10% FBS at 37°C in a humidified incubator under 5% CO_2_.

### Cell Viability

The effect of licochalcone A on cell viability was determined using the CCK8 assay. mMECs were treated with licochalcone A (1.2, 1.8, 2.4, and 3 μg/mL), LPS (1 μg/mL) or LPS+ licochalcone A (1.2, 1.8, 2.4, and 3 μg/mL) for 4 h. Subsequently, 10 μL CCK8 was added to each well. After 1 h, absorbance (OD) was measured at 450 nm on a microplate reader (Bio-Rad, CA, USA).

### Animal Experiments

BALB/c mice (8~10 weeks old, 25~30 g weight) were purchased from the Center of Experimental Animals of Baiqiuen Medical College of Jilin University (Jilin, China). Animals were housed in certified, standard laboratory cages and administered food and water *ad libitum* before experimental use. All animals care and experimental procedures were conducted in accordance with the guidelines established by the Jilin University Institutional Animal Care and Use Committee (approved on 27 February 2015, Protocol No. 2015047), designated “Guide for the Care and Use of Laboratory Animals” and approved by the Institutional Animal Care and use Committee of the University of Arizona. Animal studies were performed in compliance with arrive guidelines (26, 27).

### Animal Experimental Design

Lactating mice (5~7 days after birth of offspring) were randomly divided into five groups: control (*n* = 10), LPS (0.2 mg/mL, 50 μL) treatment (*n* = 10), LPS + licochalcone A (5 mg/kg) (*n* = 10), LPS + licochalcone A (10 mg/kg) (*n* = 10), and LPS + licochalcone A (15 mg/kg) (*n* = 10). On day seven of lactation, the control group was injected with normal saline and the three treatment groups were injected different concentrations of licochalcone A (5, 10, and 15 mg/kg). After 1 h, LPS was injected into the fourth inguinal mammary gland of mice. After 12 h, the licochalcone A treatment group was injected with the same dose of different concentrations of licochalcone A. At 24 h after LPS injection, mice were anesthetized with pentobarbital sodium (45 mg/kg) and the mammary glands were collected.

### Evaluation of Histological Changes

For histological analysis of the mammary gland, mice were euthanized and the four pairs of mammary glands fixed in 4% paraformaldehyde, followed by dehydration with ethanol. After paraffin embedding, 5 μm sections were cut and stained with hematoxylin and eosin (H&E) according to a previously described protocol (28). H&E-stained sections were examined under a light microscope to evaluate pathological changes. Simultaneously, a standard assessment method was conducted to determine mammary gland injury. Overall mammary gland injury was scored based on edema, neutrophil infiltration and hemorrhage, and three visual fields observed for each slice. Studies were performed in a blinded manner. Injury scores were representative of severity (0, no damage; 1, mild damage; 2, moderate damage; 3, severe damage; 4, very severe damage).

### ELISA for TNF-α, IL-6, and IL-1β

The protein levels of TNF-α, IL-6, and IL-1β in mammary glands were determined by ELISA kits according to the manufacturer's instructions.

### Myeloperoxidase (MPO) Activity Assay

Mammary glands were collected and weighed, followed by grinding in 4,000 μL 0.5% hexadecyl trimethyl ammonium chloride × different weights of mammary gland for 7 min (50 times per min) in a lapping instrument. After grinding, samples were centrifuged for 20 min at 12,000 g/min. The supernatant is the sample of MPO activity assay. Each sample (75 μL) and substrate (75 μL) (3,3′,5,5′-tetramethylbenzidine, 3 mM, 8,798 μL; Resorcinol, 6 mM, 180 μL, and H_2_O_2_, 3% 2.5 μL) was added to individual wells for 3~5 min, followed by the addition of 100 μL H_2_SO_4_ (2 M) to terminate the reaction. Samples were measured for MPO activity with a microplate reader at OD_450_.

### FITC-Albumin Treatment to Evaluate Alveolar Tight Junction Permeability

To visualize alveolar tight junction permeability, untreated mammary glands, or those injected with 50 μL (0.2 mg/mL) LPS and 5, 10, or 15 mg/kg licochalcone A + LPS, were treated with FITC-conjugated albumin according to a previously described protocol (29). In brief, mice were deeply anesthetized with pentobarbital and the fourth mammary gland surgically exposed. The mammary gland was immersed in 2 mL PBS containing 3 mg/mL FITC-albumin to expose the interstitial side of alveolar epithelial cells for 10 min. After treatment with FITC-albumin, the mammary gland was washed in PBS three times and immersed in PBS containing 4% paraformaldehyde for 15 min. The pre-fixed mammary gland was embedded in optimal cutting temperature (OCT) compound and frozen in liquid nitrogen after which 5 μm cryosections were obtained. Cryosections were stained with 4',6-diamidino-2-phenylindole (DAPI) and images observed under a fluorescence microscope (TCS SP5; Leica, Mannheim, Germany).

### Immunofluorescence Assays

As above, the mammary gland was embedded in OCT compound and frozen in liquid nitrogen to acquire 5 μm cryosections. Cryosections of mammary glands were fixed with 4% paraformaldehyde in PBS (pH 7.4) for 40 min at room temperature, and then incubated with PBS containing 5% donkey serum albumin to block non-specific interactions. After blocking, the sections were treated with primary antibodies diluted in 5% donkey serum albumin solution overnight at 4°C, and then washed with PBS three times for 5 min each. After washed with PBS, the sections were exposed to secondary antibodies diluted in 5% donkey serum albumin solution for 1 h at room temperature. Controls were treated in a similar manner, except that primary antibodies were excluded. Finally, all sections were washed three times with PBS and stained with DAPI for 5 min. Images of stained sections were obtained under a fluorescence microscope.

### qRT-PCR Analysis

Total RNA was isolated from cultured mMECs with TRIzol reagent (Invitrogen, Carlsbad, CA, USA) and amplification reactions conducted to detect gene levels of *TNF-*α*, IL-6, and IL-1*β. The primer sequences are shown in Table 1.

**Table 1:** The primer sequences of *TNF-*α*, IL-1*β, *IL-6* and β*-actin*.

**Item**	**Primer**	**Length (bp)**
*TNF-α* (sense)	5′-ACGGCATGGATCTCAAAGAC-3′	116
*TNF-α* (anti-sense)	5′-GTGGGTGAGGAGCACGTAGT-3′	
*IL-1β* (sense)	5′-GCTGCTTCCAAACCTTTGAC-3′	121
*IL-1β* (anti-sense)	5′-AGCTTCTCCACAGCCACAAT-3′	
*IL-6* (sense)	5′-CCGGAGAGGAGACTTCACAG-3′	134
*IL-6* (anti-sense)	5′- CAGAATTGCCATTGCACAAC-3′	
*β-actin* (sense)	5′-GTCAGGTCATCACTATCGGCAAT-3′	147
*β-actin* (anti-sense)	5′-AGAGGTCTTTACGGATGTCAACGT-3′	

### Western Blot

Total proteins were isolated from mMECs and mouse mammary glands with RIPA lysis buffer (Beyotime, Shanghai, China) (50 mM Tris, pH 7.4; 150 mM NaCl; 1% Triton X-100; 1% sodium deoxycholate; 0.1% SDS; sodium orthovanadate; sodium fluoride; EDTA, leupeptin; 1 mM PMSF). Tissue lysates were centrifuged at 12,000 g for 5 min at 4°C and protein concentrations determined with a Pierce^TM^ BCA Protein Assay Kit (Thermo scientific, China). Equal amounts of cell (25 μg)/mammary gland (60 μg) extracts were subjected to electrophoresis via 12% sodium dodecyl sulfate-polyacrylamide (SDS-PAGE) and subsequently transferred to PVDF membranes (Millipore, Darmstadt, Germany) for antibody blotting. Membranes were incubated with primary antibodies (1:1,000 dilution) at 4°C overnight, followed by HRP-conjugated goat anti-mouse (1:3,000) or goat anti-rabbit secondary antibody (1:3,000) at room temperature for 1 h. Protein bands were visualized using a Beyo Enhanced Chemiluminescence reagent kit (Beyotime, Shanghai, China) according to the manufacturer's instructions.

### Statistical Analysis

Images were generated using GraphPad prism software (La Jolla, CA, USA). Animals were randomly assigned to groups. In mouse studies, histological analysis was conducted in a blind design. In cases where overall *F*-tests were significant (*P* < 0.05), *post hoc* comparisons using Tukey's method of adjustment were conducted to determine the location of significant pairwise differences. Analyses were performed using GraphPad prism 7.00 software.

## Results

### Licochalcone A Administration Alleviates Pathological Injury of Mammary Gland in LPS-Induced Mice Mastitis

**Figure 2:**
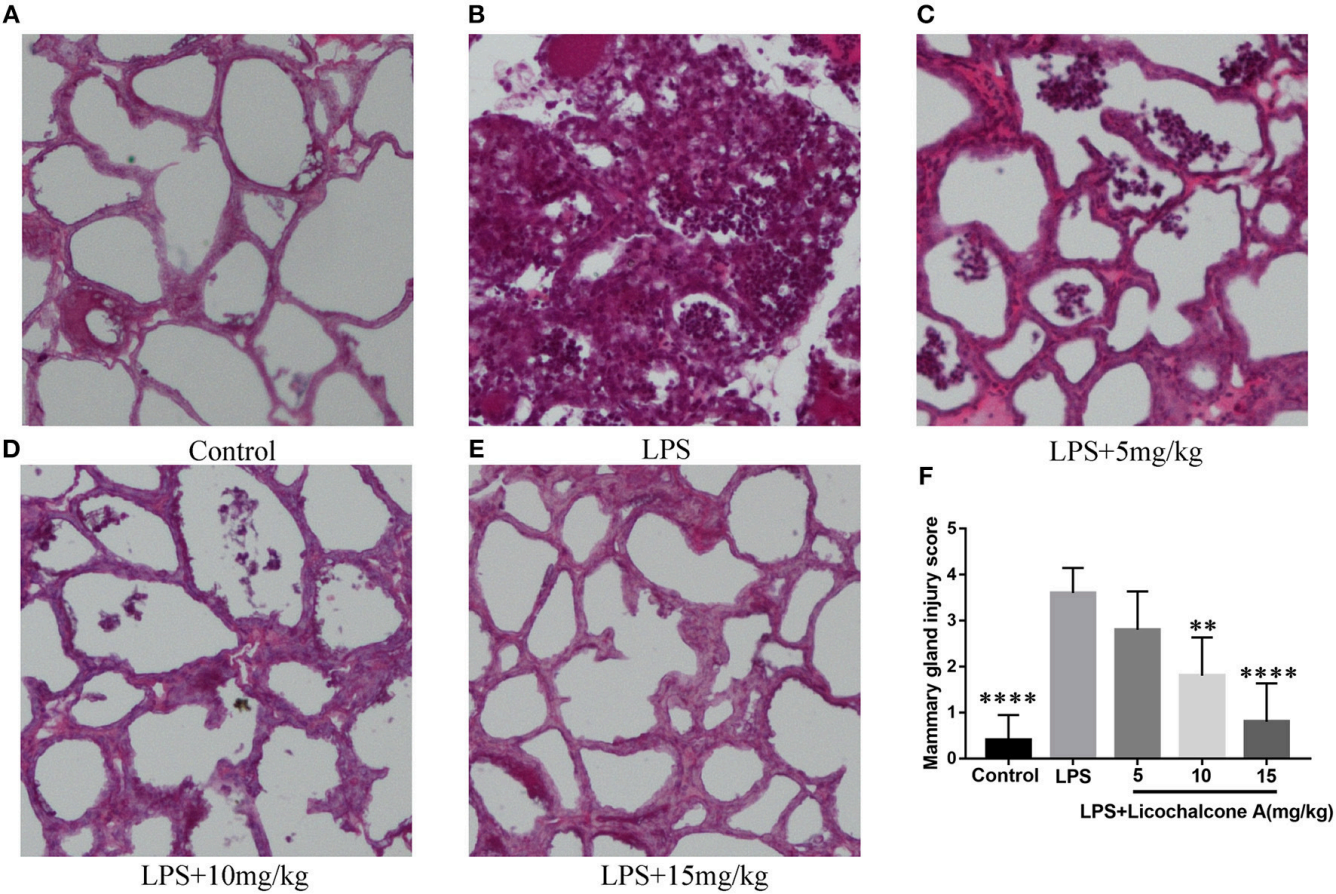
Effects of licochalcone A on pathological injury of mammary gland in LPS-induced mice mastitis. Mammary gland tissues from each experimental group (*n* = 10) were obtained at 24 h after LPS administration, sectioned and stained with H&E (original magnification 100 ×). Mammary gland tissues of **(A)** control group, **(B)** LPS group, **(C)** LPS + licochalcone A (5 mg/kg) group, **(D)** LPS + licochalcone A (10 mg/kg) group, and **(E)** LPS + licochalcone A (15 mg/kg) group. **(F)** Mean injury scores of mammary glands were determined according to a previously described three-point scale. Values are presented as means ± *SD* (**p < 0.01 and **** *p* < 0.0001 vs. LPS group).

To study the protective effect of licochalcone A on mastitis, the mammary glands were collected, sectioned and stained with H&E (Figure 2). There were no pathological injuries of mammary gland from control group mice (Figure 2A). Compared with control group, mammary gland from LPS group presented severe pathological injuries, such as thickening of the alveolar wall, inflammatory cell infiltration (Figure 2B). However, these pathological injuries were alleviated by Licochalcone A in a dose-dependent manner (Figures 2C–F, Supplementary Figure 3).

### Licochalcone A Inhibits MPO Activity in LPS-Induced Mice Mastitis

**Figure 3:**
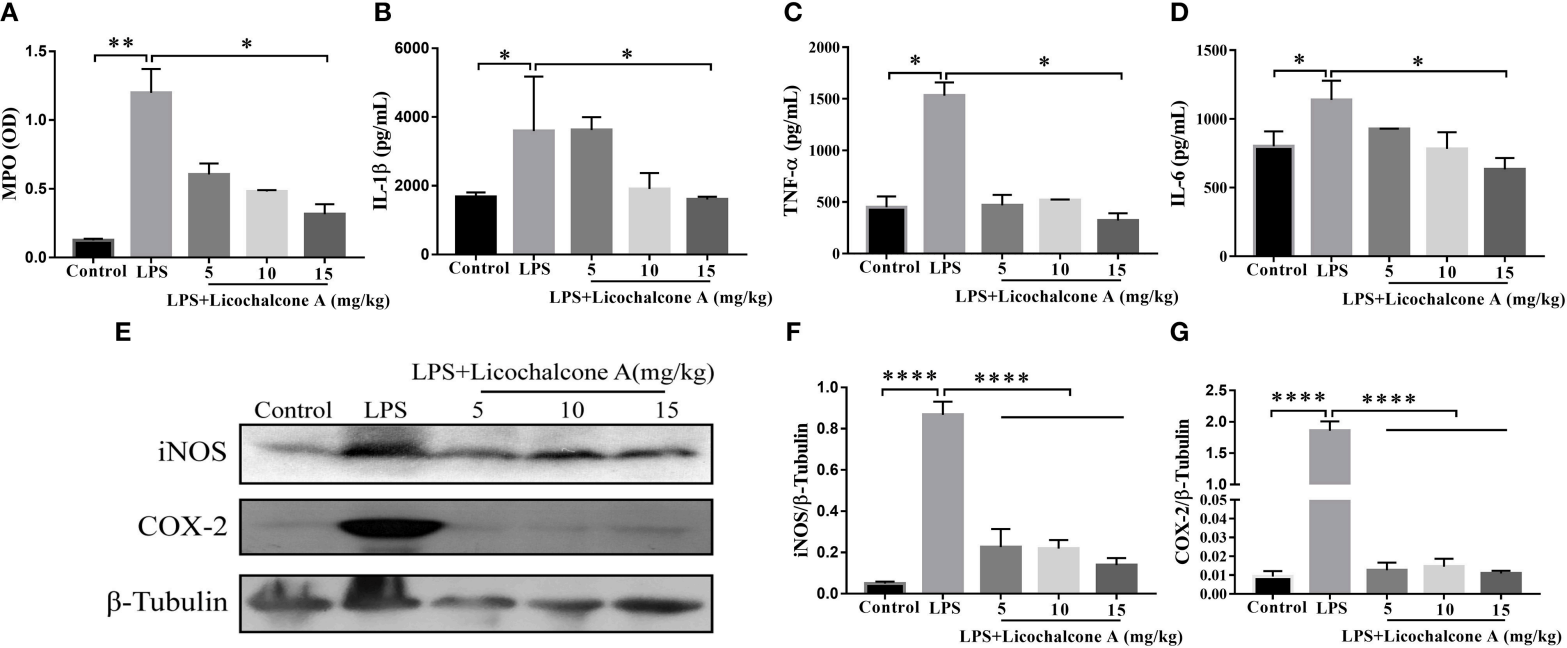
Effects of Licochalcone A on inflammatory response in LPS-induced mice mastitis. Mammary gland tissues from each experimental group (*n* = 10) were obtained at 24 h after LPS administration. **(A)** Myeloperoxidase (MPO) activity assay. The protein levels of IL-1β **(B)**, TNF-α **(C)**, and IL-6 **(D)** were detected using ELISA. Western blot assay of inducible nitric oxide synthase (iNOS) **(E,F)** and cyclooxygenase-2 (COX-2) **(E,G)**, and the relative protein levels were quantified by scanning densitometry and normalized to β-tubulin. Values are presented as means ± *SD* (*p < 0.05, **p < 0.01, and ****p < 0.0001 vs. LPS group).

Neutrophils are the first line of innate immune defense against infection. The accumulation of neutrophil in mammary gland reflected the level of inflammation. In order to detect the degree of inflammation, the activity of MPO, which is a marker of neutrophil, was detected. The result found that LPS injection significantly increased MPO activity in mammary gland tissues compared with control group (Figure 3A). Compared with LPS group, licochalcone A treatment remarkably reduced the MPO activity in a dose-dependent manner (Figure 3A).

### Licochalcone A Reduces Pro-Inflammatory Mediators Production in LPS-Induced Mice Mastitis

Pro-inflammatory cytokines play important roles in the development of inflammation. To elucidate the effect of licochalcone A on the level of inflammation in LPS-induced mice mastitis, the production of the three major pro-inflammatory cytokines (including IL-6, IL-1β, and TNF-α) in mammary gland tissues were detected by ELISA. As shown in Figure 3, LPS significantly increased the protein levels of IL-6 (Figure 3B), IL-1β (Figure 3C), and TNF-α (Figure 3D), and licochalcone A treatment suppressed this effect in mammary gland. iNOS and COX-2 are two important pro-inflammatory proteins correlated with inflammation. In our experiments, the protein levels of iNOS and COX-2 were also examined by western blot. The result found that LPS injection leading to remarkably production of iNOS (Figures 3E,F) and COX-2 (Figures 3E,G), and licochalcone A administration significantly inhibits LPS-induced production of iNOS and COX-2 in mammary gland.

### Licochalcone A Inhibits the AKT/NF-κB and Mitogen-Activated Protein Kinase (MAPK) Signaling Pathways in LPS-Induced Mice Mastitis

**Figure 4:**
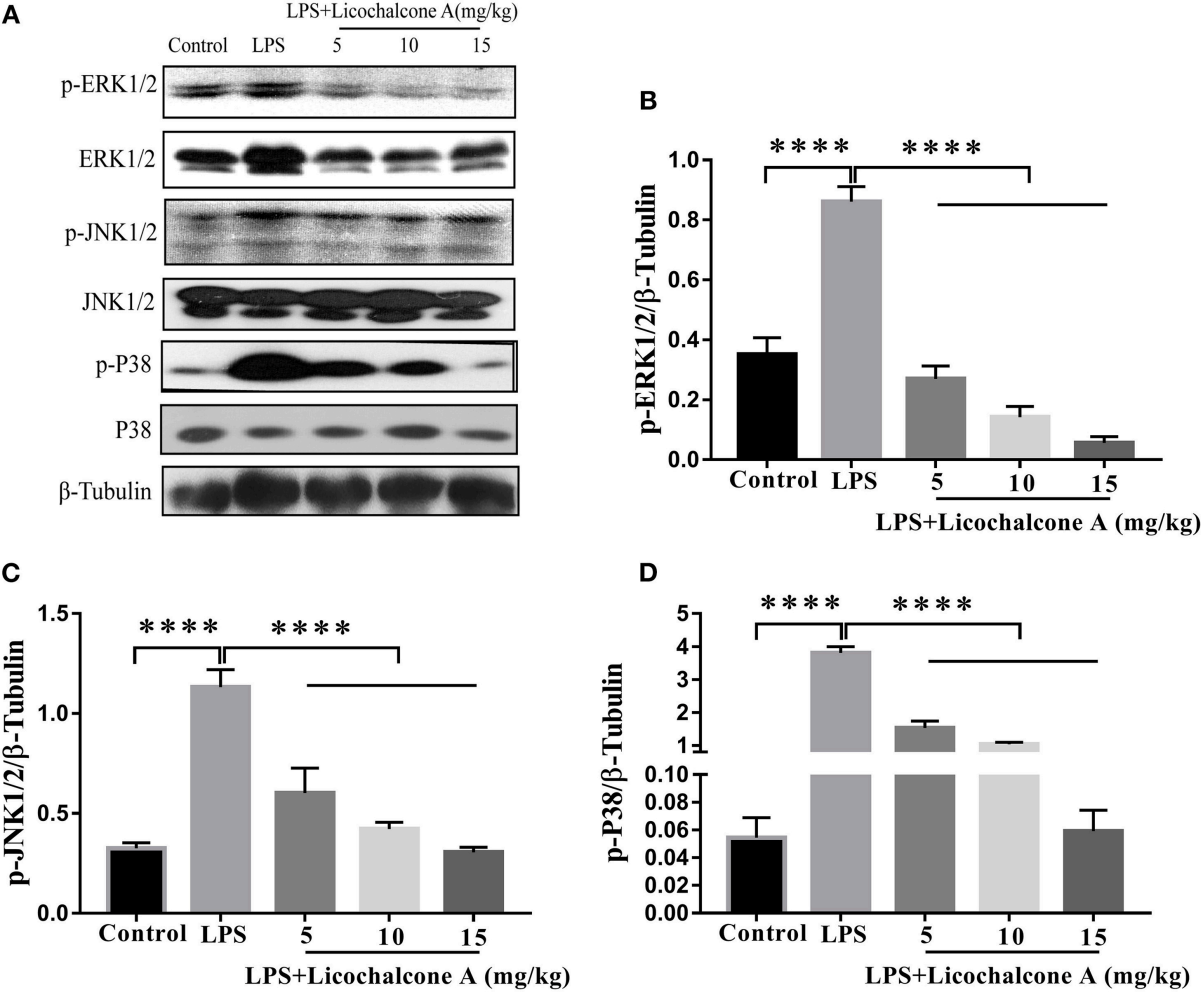
Effects of licochalcone A on mitogen-activated protein kinase (MAPK) signaling pathway in LPS-induced mice mastitis. Mammary gland tissues from different experimental groups were obtained 24 h after LPS administration. The tissue lysates were prepared and subjected to western blot by using p-ERK1/2 **(A,B)**, p-JNK1/2 **(A,C)**, p-P38 **(A,D)** antibodies, respectively. Each immunoreactive band was digitized and expressed as a ratio of the β-tubulin level. Values are presented as means ± *SD* (****p < 0.0001 vs. LPS group).

**Figure 5:**
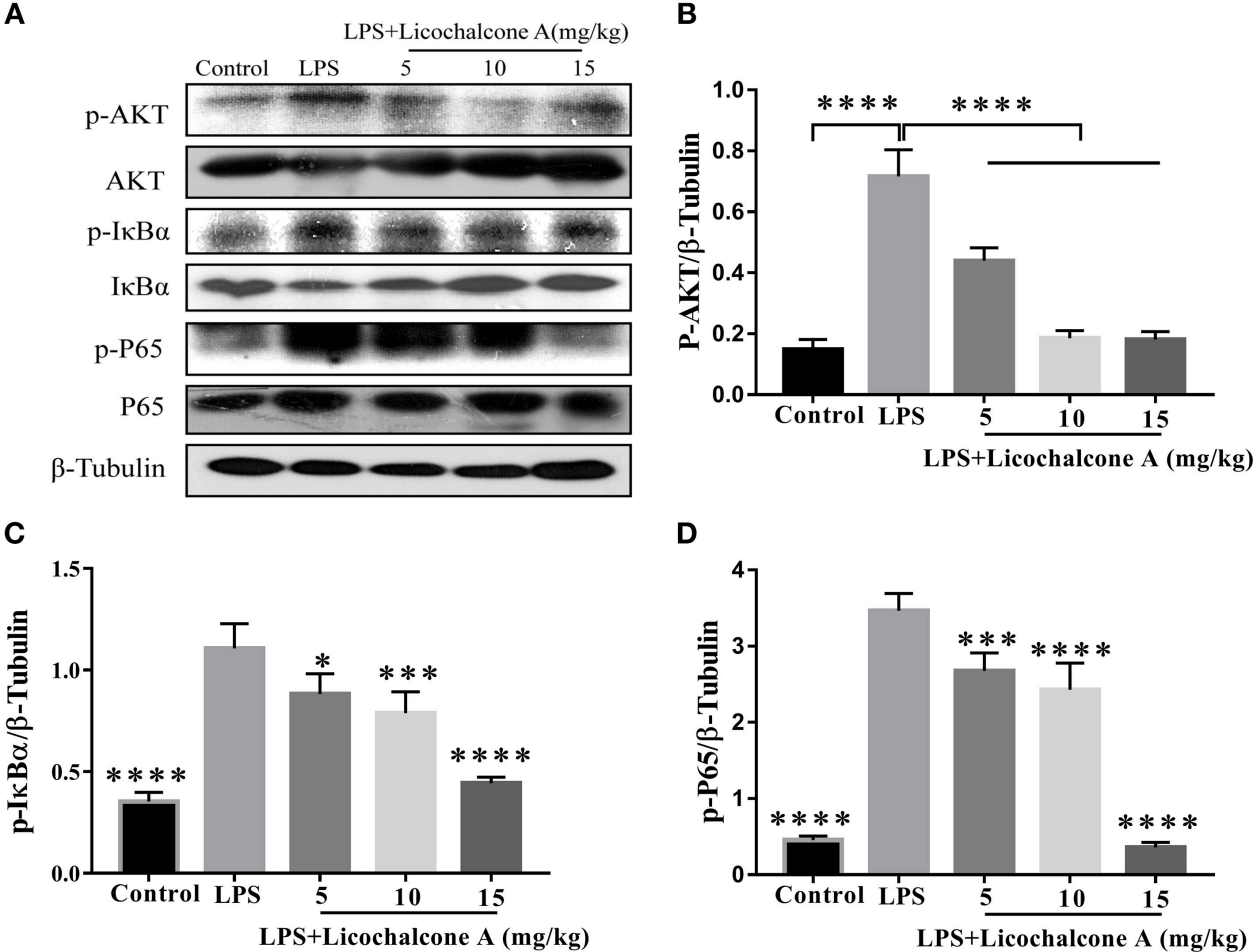
Effects of licochalcone A on AKT/NF-κB signaling pathway in LPS-induced mice mastitis. Mammary gland tissues from different experimental groups were obtained 24 h after LPS administration and total protein. The tissue lysates were prepared and subjected to western blot by using p-AKT **(A,B)**, p-IκBα **(A,C)**, and p-P65 **(A,D)** antibodies, respectively. Each immunoreactive band was digitized and expressed as a ratio of the β-tubulin level. Values are presented as means ± *SD* (*p < 0.05, ***p < 0.001, and ****p < 0.0001 vs. LPS group).

AKT/NF-κB and MAPK signaling pathways are known to play important role in the regulation of inflammatory mediator production. To elucidate the anti-inflammatory mechanism, the activation of AKT/NF-κB and MAPK signaling pathways were detected by western blot. The result found that the LPS injection significantly increased the phosphorylation of ERK1/2 (Figures 4A,B), JNK1/2 (Figures 4A,C), P38 (Figures 4A,D), AKT (Figures 5A,B), IκBα (Figures 5A,C), and P65(Figures 5A,D) compared with control group. Moreover, licochalcone A treatment significantly inhibited LPS-induced phosphorylation of ERK1/2 (Figures 4A,B), JNK1/2 (Figures 4A,C), P38 (Figures 4A,D), AKT (Figures 5A,B), IκBα (Figures 5A,C), and P65 (Figures 5A,D) in a dose-dependent manner.

### Licochalcone A Repairs the Blood–Milk Barrier Integrity in LPS-Induced Mice Mastitis

**Figure 6:**
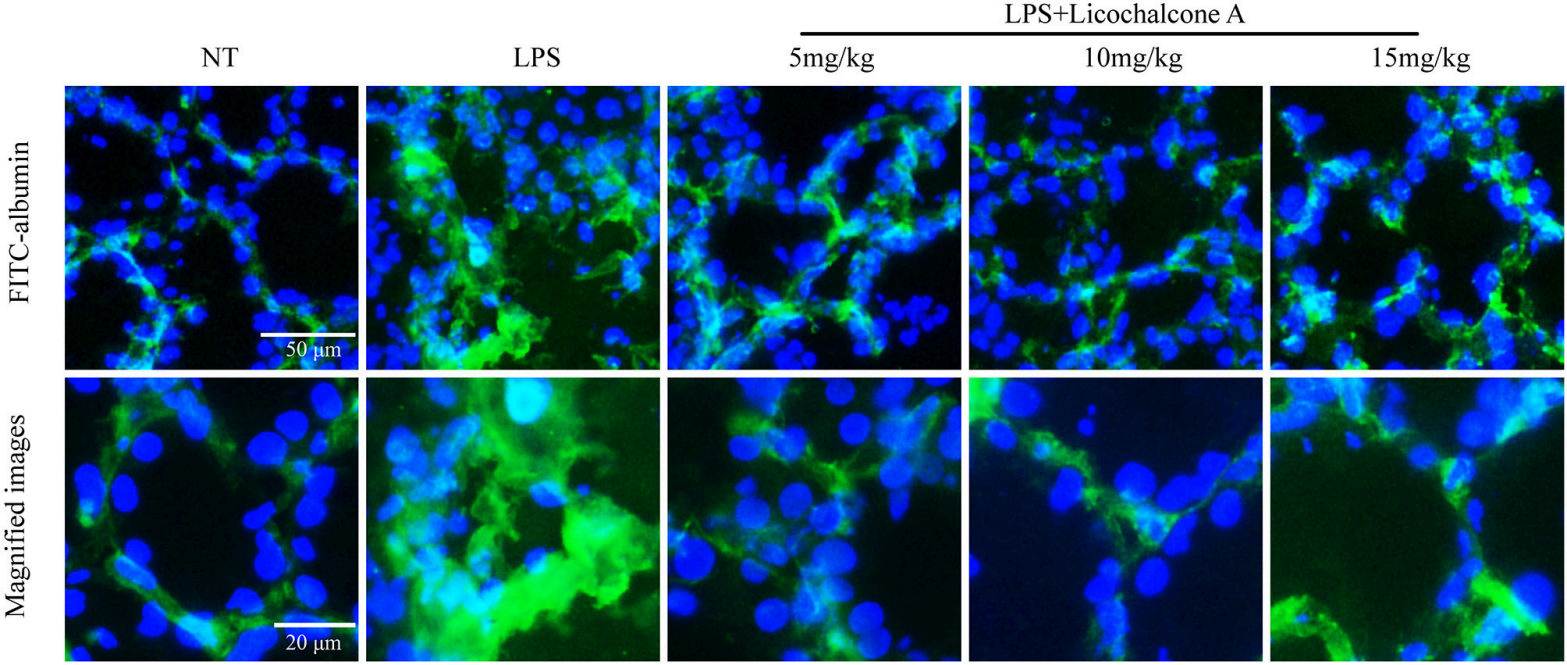
Effects of licochalcone A on the permeability of the alveolar epithelium. Mammary gland tissues from different experimental groups were obtained 24 h after LPS administration and immersed in FITC-albumin containing 0.5 mM CaCl_2_ and 0.5 mM MgCl_2_-containing phosphate-buffered saline (mPBS), and localization of FITC-albumin observed after cutting frozen sections. Green and blue colors represent FITC-albumin and nuclei (4′,6-diamidino-2-phenylindole, DAPI), respectively. FITC-albumin was observed in the alveolar lumen of LPS-injected mammary glands.

Blood–milk barrier, a specific structure of mammary gland, which could restrict the invasion of microbial. In order to elucidate the effect of licochalcone A on blood-milk barrier, the leakage of FITC-albumin from the interstitial side into alveolar lumen was determined. The result found that FITC-albumin was distributed in the interstitial side in the NT group, and FITC-albumin permeated into alveolar lumen in the LPS group (Figure 6). As expect, licochalcone A could inhibit LPS-induced permeation of FITC-albumin (Figure 6).

### Licochalcone A Enhances Protein Levels of Tight Junction Proteins and Restores Localization Changes of Claudin-3 and Occludin in LPS-Induced Mice Mastitis

**Figure 7:**
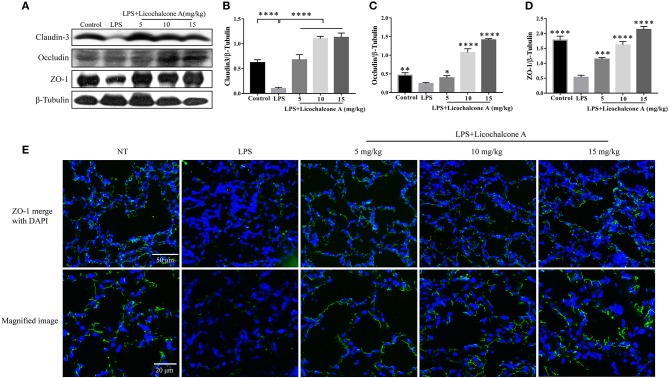
Protein levels change in claudin-3, occluding, and ZO-1 after LPS or LPS + licochalcone A injection. Protein levels was measured using ImageJ software (http://imagej.nih.gov/ij/) and normalized to that of β-tubulin. **(A–D)** Results of western blot analysis of claudin-3, occludin, ZO-1, and β-tubulin in mammary glands after LPS and LPS + licochalcone A injection. **(B–D)** Protein levels of claudin-3, occludin and ZO-1 normalized to that of β-tubulin. **(E)** Immunostaining images of ZO-1 (green) and nuclear staining with DAPI (blue) in mammary glands treated with LPS and licochalcone A. Values are presented as means ± *SD* (*n* = 10 in each group) (*p < 0.05, **p < 0.01, ***p < 0.001, and ****p < 0.0001 vs. LPS group).

**Figure 8:**
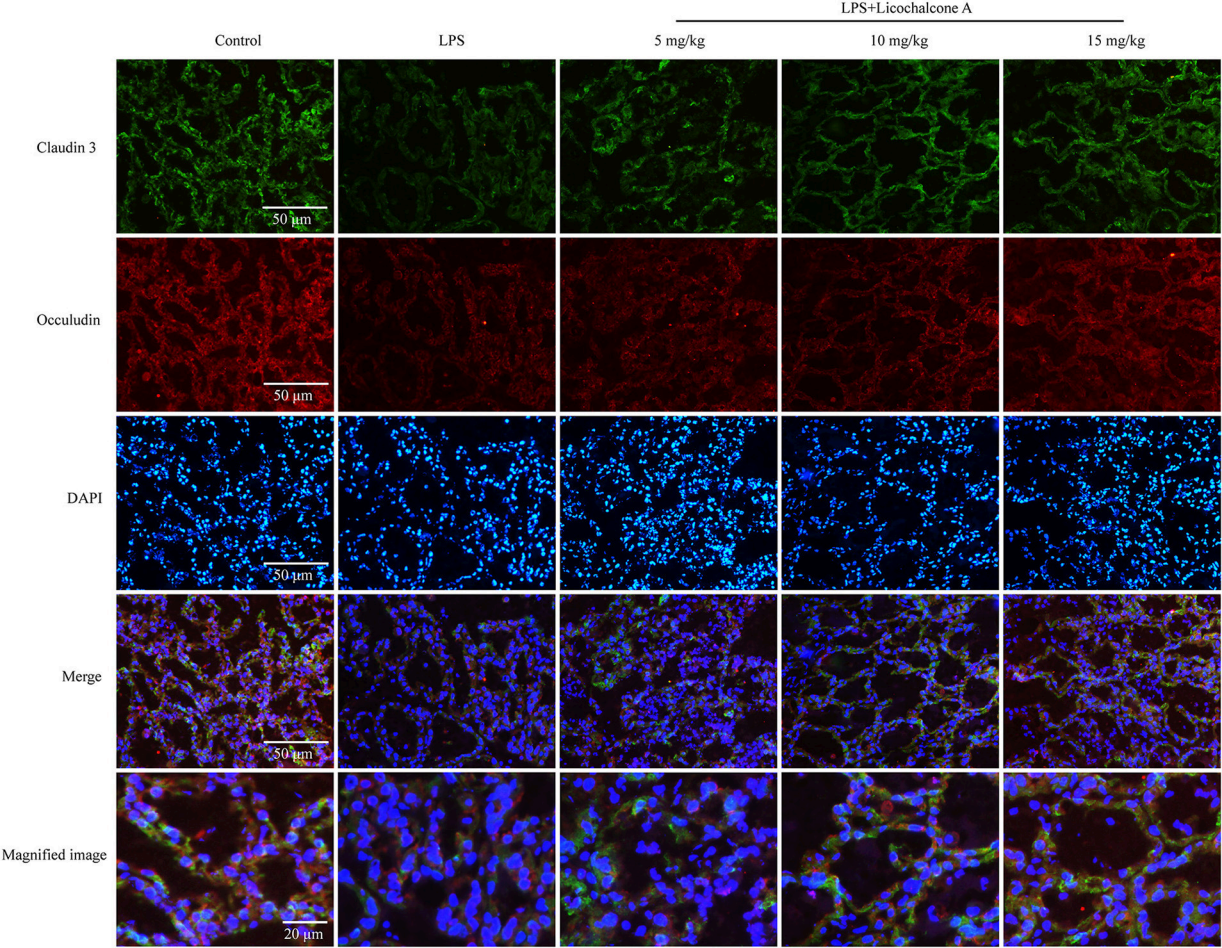
Immunostaining images of claudin-3 (green), occludin (red) and nuclear staining with DAPI (blue) in mammary glands treated with LPS and licochalcone A. Weak fluorescence intensity was observed for claudin-3 and occludin in the LPS group. With increasing doses, the fluorescence intensities of claudin-3 and occludin were gradually increased.

Tight junction, the basic structure of blood-milk barrier, which could restrict the paracellular flow of aqueous molecules, ions, water, and microbes. To clarify the mechanism of licochalcone A on repairing the blood–milk barrier integrity, the protein levels tight junction components (including ZO-1, Occludin, and claudin3) and the localization changes of claudin-3 and occludin were determined by western blot and immunofluorescence assays. Protein levels of claudin-3, occludin and ZO-1 in the LPS group were significantly lower than those in the LPS + licochalcone A group (Figures 7A–D). Immunofluorescence staining revealed significantly lower fluorescence intensities in the LPS group, compared to the LPS + licochalcone A groups (Figure 7E), suggesting that licochalcone A repairs the tight junction destroyed by LPS. Weaker fluorescence intensities of claudin-3 and occludin were observed in the LPS group, compared to the control and LPS + licochalcone A groups. Claudin-3 localized with occludin at the apical-most regions in mammary epithelial cells (MECs) before LPS injection with no differences between localization patterns (Figure 8). At 24 h after LPS injection, claudin-3 mainly localized with occludin at the apical-most regions. Single localization of claudin-3 without occludin was also observed around these areas. In the presence of increasing doses of licochalcone A, claudin-3 re-localized with occludin at the apical-most regions, suggesting that licochalcone A increases the protein levels of tight junction and restores the original localization patterns of claudin-3 and occludin.

### Licochalcone A Inhibits LPS-Induced Inflammatory Response in mMECs

**Figure 9:**
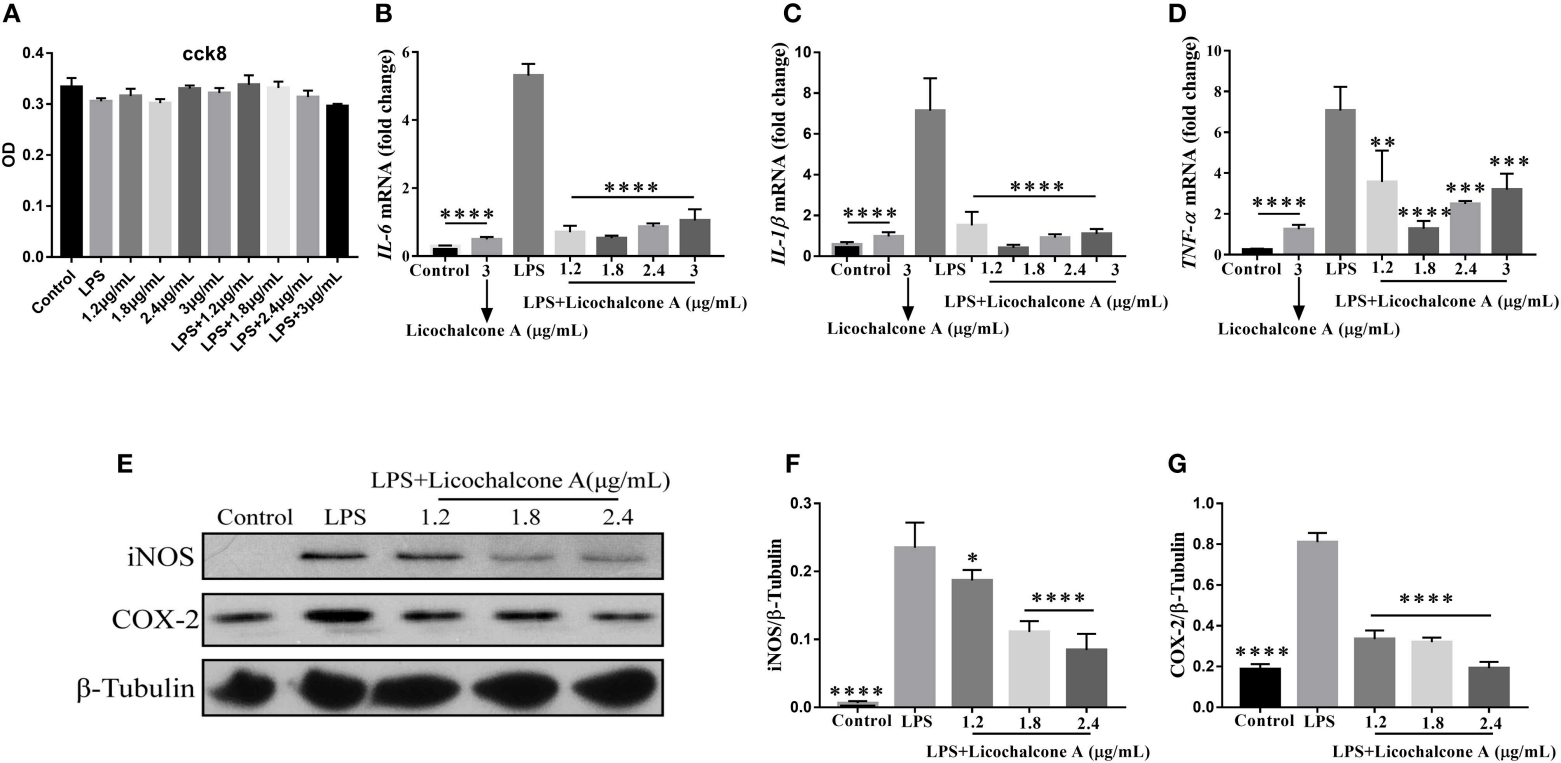
Effects of Licochalcone A on LPS-induced inflammatory response in mouse mammary epithelial cells (mMECs). Cells were cultured with different concentrations of licochalcone A (1.2, 1.8, 2.4, and 3 μg/mL) for 4 h and viability determined with the CCK8 assay. **(A)** The effect of licochalcone A and licochalcone A + LPS were determined by CCK8 assay. mMECs were pretreated with Licochalcone A (1.2, 1.8, 2.4, and 3 μg/mL) for 1 h and then stimulated with LPS for 4 h, protein and mRNA levels were determined by qRT-PCR and western blot. The mRNA levels of *IL-6* **(B)***, IL-1*β **(C)**, and *TNF-*α **(D)**, and the relative mRNA level was normalized to β*-actin* mRNA. The protein levels of COX-2 **(E,G)** and iNOS **(E,F)**, and the relative protein levels were quantified by scanning densitometry and normalized to β-tubulin. Values are presented as means ± *SD* (*n* = 3) (*p < 0.05, **p < 0.01, ***p < 0.001, and ****p < 0.0001 vs. LPS group).

In our *in vivo* experiments, we have proven that licochalcone A have anti-inflammatory role in LPS-induced mice mastitis. To further this function of licochalcone A, the effect of licochalcone A on LPS-induced inflammatory response in mMECs was examined. Firstly, the cytotoxicity of licochalcone A, LPS and licochalcone A + LPS on mMECs was determined; The result found that licochalcone A, LPS and licochalcone A + LPS have no cytotoxicity on mMECs (Figure 9A). Secondly, mMECs were pretreated with licochalcone A (1.2, 1.8, 2.4, and 3 μg/mL) for 1 h and then stimulated with LPS for 4 h, the mRNA levels of IL-6, IL-1β and TNF-α were determined by qRT-PCR, and the protein levels of COX2 and iNOS were determined by western blot. The result of qRT-PCR shown that LPS significantly increased the mRNA levels of IL-6, IL-1β, and TNF-α compared with control and licochalcone A (3 μg/mL) treatment groups, and licochalcone A notably inhibited the increased mRNA levels of IL-6, IL-1β, and TNF-α (Figures 9B–D). The western result found that licochalcone A significantly inhibited LPS-induced production of COX2 and iNOS in a dose-dependent manner (Figures 9E–G). These results are consistent with those *in vivo*, which further confirms the anti-inflammatory effect of licochalcone A.

### Licochalcone A Inhibits LPS-Induced Activation of AKT/NF-κB and MAPK Signaling Pathways in mMECs

**Figure 10:**
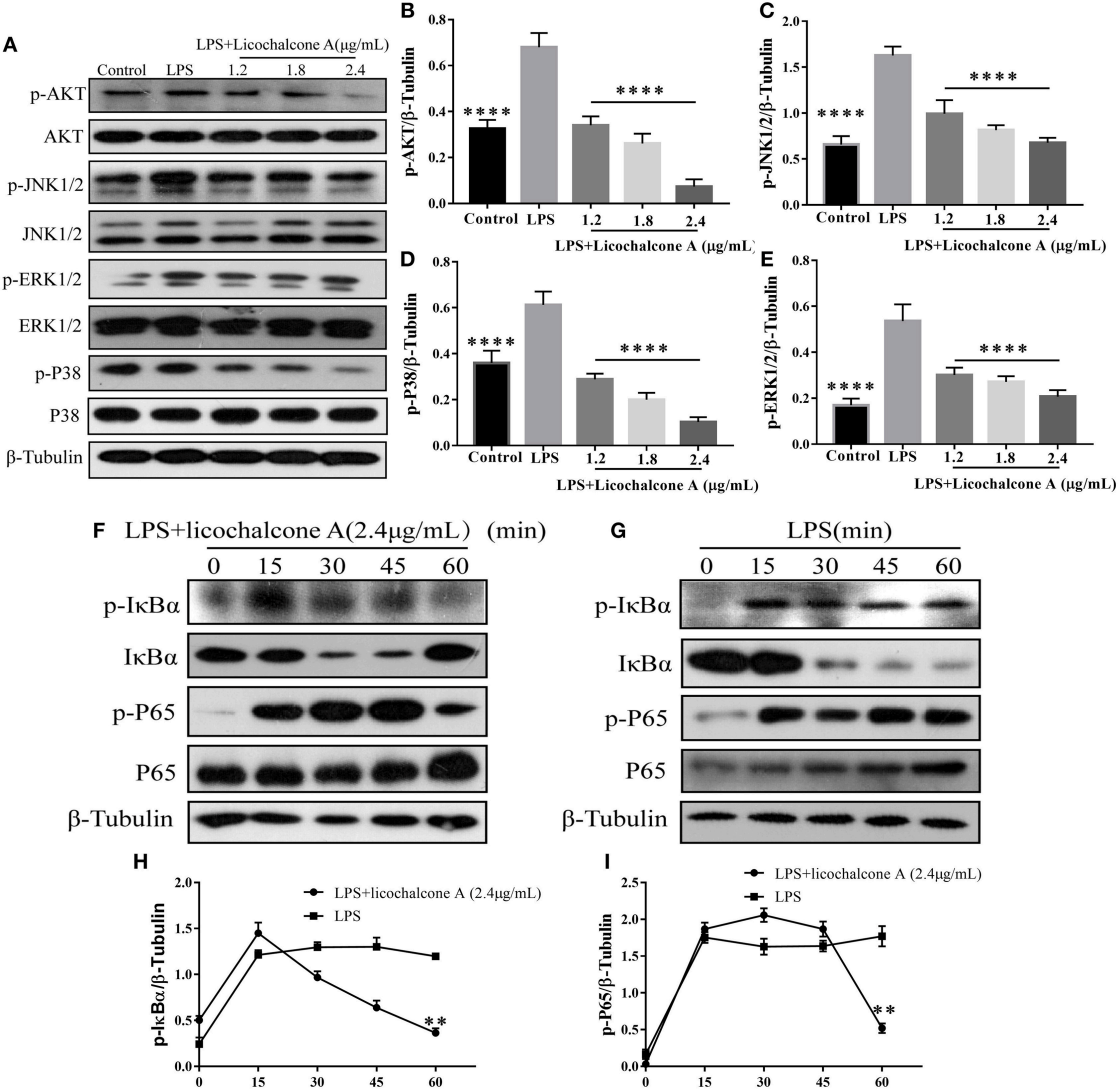
Effects of licochalcone A on LPS-induced activation of AKT/NF-κB and MAPK signaling pathways in mMECs. **(A–E)** Total protein in mMECs was collected after 4 h of LPS stimulation. Licochalcone A was added 1 h before LPS stimulation. Protein levels of p-AKT, p-JNK1/2, p-ERK1/2, and p-P38 were detected via western blot and quantitatively assessed via densitometry using β-tubulin as an internal control. Protein levels were measured using ImageJ software (http://imagej.nih.gov/ij/) and normalized to that of β-tubulin. **(F–I)** MMECs were divided into LPS (1 μg/mL) or LPS + licochalcone A (2.4 μg/mL) groups. After adding LPS to serum-free medium for 1 h, licochalcone A was added. Protein was collected after 0, 15, 30, 45, and 60 min. **(F)** Western blot analysis of p-IκBα, IκBα, p-P65, and P65 in mMECs were treated with LPS + licochalcone A. **(G)** Western blot analysis of p-IκBα, IκBα, p-P65, and P65 in mMECs were treated with LPS only. **(H–I)** The phosphorylation of P65 and IκBα at different time-points under LPS and LPS + licochalcone A treatment were detected via western blot. Values are presented as means ± *SD* (*n* = 3) (**p < 0.01 vs. LPS, ****p < 0.0001 vs. LPS).

To further elucidate the anti-inflammatory mechanism, we also examined the effect of licochalcone A on LPS-induced activation of AKT/NF-κB and MAPK signaling pathways in mMECs. Firstly, mMECs were pretreated with licochalcone A (1.2, 1.8, and 2.4 μg/mL) for 1 h and then stimulated with LPS for 4 h, the phosphorylations of AKT, ERK1/2, JNK1/2, and P38 were determined by western blot. The result found that licochalcone A inhibited LPS-induced phosphorylations of AKT (Figures 10A,B), ERK1/2 (Figures 10A,E), JNK1/2 (Figures 10A,C), and P38 (Figures 10A,D) in a dose-dependent manner. Secondly, mMECs were pretreated with licochalcone A (2.4 μg/mL) for 1 h and then stimulated with LPS for 15, 30, 45, and 60 min, the phosphorylation of IκBα and NF-κB examined by western blot. With increasing time-periods, the NF-κB pathway was more strongly activated in the LPS group. Conversely, in the LPS + licochalcone A group, the NF-κB pathway was gradually suppressed, implying that inhibition of inflammation in mMECs by licochalcone A is time-dependent (Figures 10F–I).

### Licochalcone A Enhances the Protein Levels of ZO-1, Occludin, and Claudin3 in mMECs

**Figure 11:**
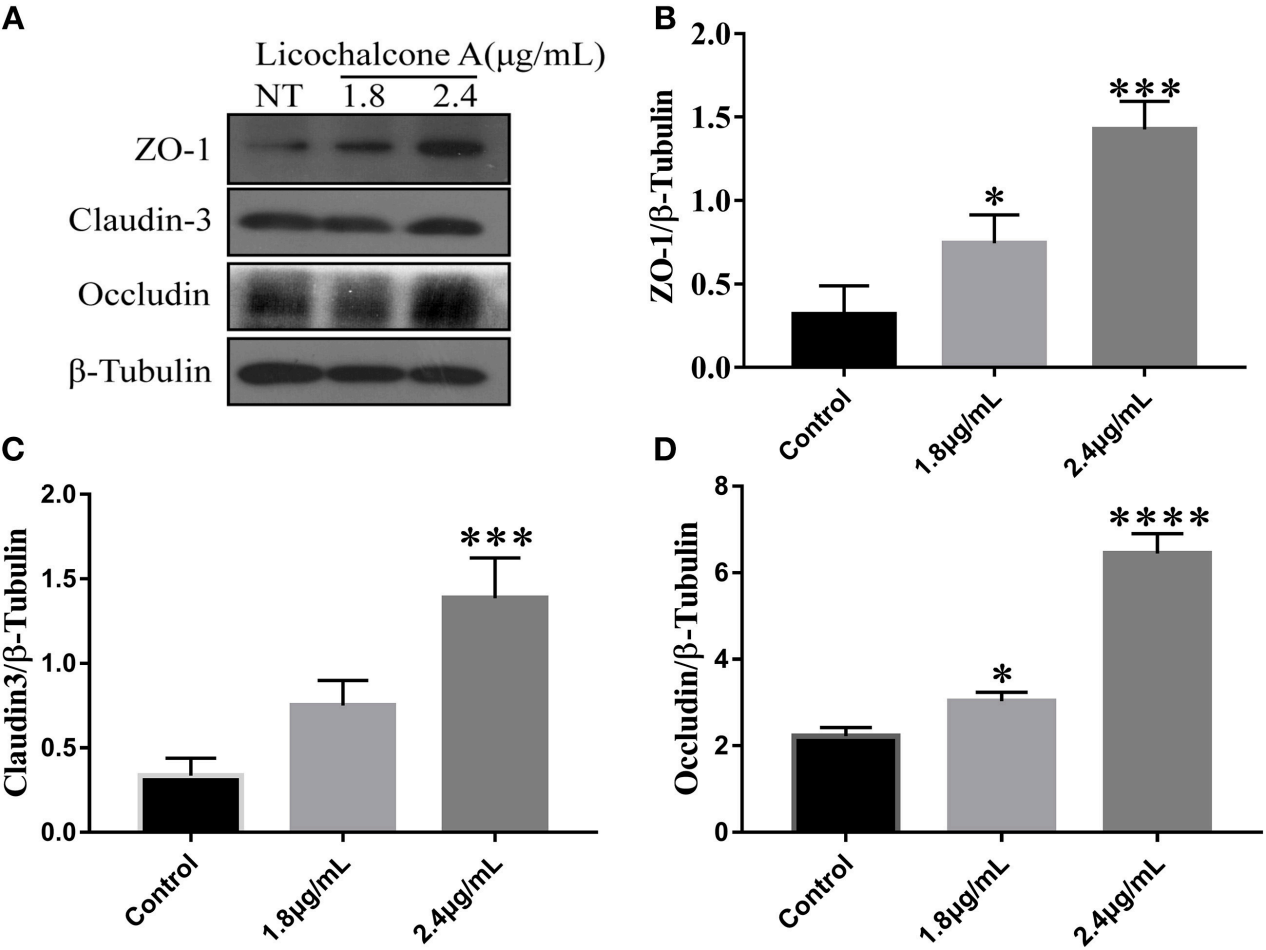
Effects of licochalcone A on the protein levels of ZO-1, Occludin, and claudin3 in mMECs. mMECs were serum-starved for 3 h before treatment with Licochalcone A (1.8, 2.4 μg/mL) for 24 h **(A)**. The protein levels of ZO-1 **(B)**, claudin3 **(C)**, and Occludin **(D)** were determined by western blot, and the relative protein levels were quantified by scanning densitometry and normalized to β-tubulin. Values are presented as means ± *SD* (*n* = 3) (**p < 0.01, ***p < 0.001 and ****p < 0.0001 vs. Control group).

In our *in vivo* experiments, we have found that licochalcone A repaired blood-milk barrier integrity by increasing the protein levels of tight junction components (including ZO-1, Occludin, and claudin3). To further clarify whether licochalcone A increase the protein levels of tight junction components, mMECs were treated with 2.4 μg/mL for 24 h, and the protein levels of including ZO-1, Occludin, and claudin3 were detected by western blot. As shown in Figure 11, licochalcone A remarkably increased the protein levels of ZO-1 (Figures 11A–D), Occludin (Figures 11A–D), and claudin3 (Figures 11A–D) in dose-dependent manner.

**Figure 12:**
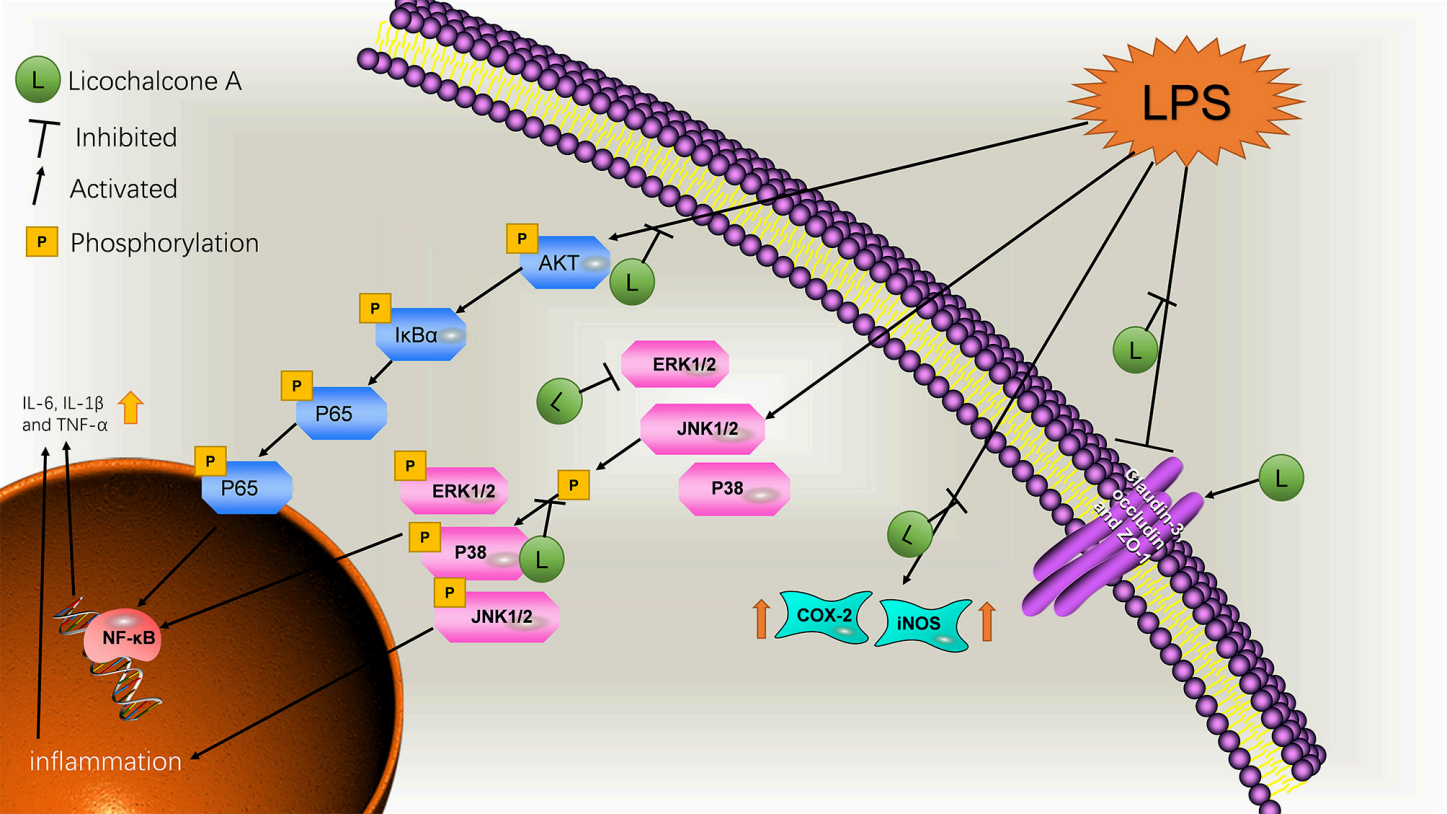
Schematic diagram of the pathways by which licochalcone A inhibits LPS-induced mastitis.

## Discussion

Mastitis is a common clinical disease in humans and animals by microbial invasion. Antibiotic therapy is the current mainstream treatment but prone to drug resistance with significant side-effects (30). Our findings demonstrated that licochalcone A could significantly alleviate mastitis via reducing inflammatory response and protecting the blood-milk barrier in LPS-induced mice mastitis. The further mechanistic study found that licochalcone A significantly suppressed the MAPK and AKT/NF-κB signaling pathways *in vivo* and *in vitro*. These data suggested that licochalcone A might be a strong candidate for treatment of mastitis.

Previous reports have shown that monomer composition could relieve mastitis, such as magnolol. Magnolol played an anti-inflammatory effect by inhibiting TLR4 (31). The traditional Chinese medicine monomer, licochalcone A, is reported to exert anti-inflammatory effects (32). Our team has found that licochalcone A could protect dopaminergic neurons via inhibiting neuro- inflammation in LPS-induced PD rat model (11). There is no report about the effect of licochalcone A on development of mastitis. Therefore, we examined the effect and the mechanism of licochalcone A on mastitis *in vivo* and *in vitro*. *In vivo*, we set up three concentration gradients, and found that the mastitis gradually relieved with the increase of licochalcone A concentration. It was also obvious from H&E staining that a large number of neutrophils were gathered in the mammary acini, and the thickening of the acinus was enhanced. As expected, licochalcone A could improve these micropathological changes. In addition, we found that licochalcone A also significantly inhibited the protein or gene levels of iNOS, COX-2, IL-6, IL-1β, and TNF-α *in vivo* and *in vitro*. At the same time, licochalcone A can also alleviate the inflammatory response of macrophages (Supplementary Figure 2). These results indicate that licochalcone A can significantly alleviate mouse mastitis.

Previous results have confirmed the anti-inflammatory effect of licochalcone A, but its anti-inflammatory mechanism has not been studied, so we studied the anti-inflammatory mechanism of licochalcone A in subsequent experiments. Interestingly, we found that licochalcone A can inhibit the phosphorylation of MAPK and AKT/NF-κB signaling pathways. Previous studies indicate that LPS triggers TLR4 downstream inflammatory pathways to stimulate the release of inflammatory cytokines (33). Two of the most important signaling pathways in inflammation are TLR4/MYD88/MAPK (Supplementary Figure 1) and AKT/NF-κB (34). The MAPK signaling pathway plays a key role in regulation of gene expression and cytoplasmic function (35). ERK1/2, JNK1/2, and P38 proteins in the MAPK signaling pathway are involved in the inflammation and stress response of the body and present crucial anti-inflammatory drug targets (36, 37). The AKT/NF-κB signaling pathway mainly activates NF-κB by phosphorylating AKT to degrade IκBα (38). However, in the MAPK signaling pathway, p-ERK1/2 and p-P38 directly activate the NF-κB signaling pathway to promote inflammation (39). *In vivo*, with a gradual increase in the licochalcone A dose, protein levels of p-ERK1/2, p-JNK1/2, and p-P38 in MAPK signaling pathway were decreased. Our *in vitro* data were consistent with those obtained *in vivo*. However, the protein level of ERK1/2 was additionally inhibited *in vivo*. Therefore, licochalcone A reduced phosphorylation of ERK1/2 by inhibiting ERK1/2 protein levels. The protein levels of ERK1/2 did not change *in vitro*, which might be due to the short stimulation time, but its phosphorylation level was also inhibited. In subsequent experiments, we found that licochalcone A also inhibited the activation of AKT/NF-κB signaling pathway. *In vivo* and *in vitro* results showed that p-AKT, p-IκBα and p-P65 levels in the LPS group were significantly increased. However, licochalcone A concentration-dependently inhibited the protein levels of p-AKT, p-IκBα, and p-P65 *in vivo* and *in vitro*. Our experiments collectively indicate that licochalcone A inhibited the expression of inflammatory cytokines via inhibiting MAPK and AKT/NF-κB signaling pathways, thereby alleviating mastitis.

Previous studies have shown that blood–milk barrier is an important barrier for mammary gland resistance to invasion by external bacteria (40). Mastitis increases blood–milk barrier permeability and disruption of the tight junction, which can further aggravate the condition (41). Claudin-3, occluding, and ZO-1 are important tight junction proteins in MECs and their destruction directly affects the tight junction between cells (42). LPS is reported to damage the tight junctions between MECs (23). In our experiment, we have studied the anti-mastitis mechanism of licochalcone A. But how did licochalcone A affect the blood-milk barrier? Interestingly, we found that FITC-albumin in LPS group could enter in mammary gland acinus, and FITC-albumin in mammary gland acinus was decreased gradually with the increase of licochalcone A dosage *in vivo*. We also found that licochalcone A could increase the protein levels of claudin-3, occludin, and ZO-1 *in vivo* and *in vitro*. These data suggested that licochalcone A improved the integrity of blood-milk barrier via increasing the protein levels of tight junction proteins. These data suggested that licochalcone A alleviated mastitis at least partially by improving the integrity of blood-milk barrier. This suggests that licochalcone A may play an important role in protecting the blood-milk barrier.

All our experiments indicate that licochalcone A reduces the expression of inflammatory factors by inhibiting the MAPK and AKT/NF-κB signaling pathway, and repairs blood–milk barrier damage caused by inflammation, supporting a novel clinical application of this compound in the treatment of mastitis.

## Author Contributions

YY, XK, QG, YL, YC, JW, DX, and HM helped with the experiments. SF, BL, and JL revised the content of the article. All authors listed have made a substantial, direct and intellectual contribution to the work, and approved it for publication.

### Conflict of Interest Statement

The authors declare that the research was conducted in the absence of any commercial or financial relationships that could be construed as a potential conflict of interest.

## Supplementary Material

Supplementary Figure 1 Effects of licochalcone A on LPS-induced activation of TLR4/MYD88 signaling pathways in mMECs. **(A–C)** Total protein in mMECs was collected after 4 h of LPS stimulation. Licochalcone A was added 1 h before LPS stimulation. Protein levels of TLR4 and MYD88 were detected via western blot and quantitatively assessed via densitometry using β-tubulin as an internal control. Protein levels were measured using ImageJ software (http://imagej.nih.gov/ij/) and normalized to that of β-tubulin. Values are presented as means ± SD (*n* = 3) (^*^*p* < 0.05, ^**^*p* < 0.01, ^***^*p* < 0.001, and ^****^*p* < 0.0001 vs. LPS group).

Supplementary Figure 2 Effects of Licochalcone A on LPS-induced inflammatory response in mouse macrophage (mMECs). The mRNA levels of IL-6 **(A)**, IL-1β **(B)**, and TNF-α **(C)**, and the relative mRNA level was normalized to β-actin mRNA. Values are presented as means ± SD (*n* = 3) (^*^*p* < 0.05, ^**^*p* < 0.01, ^***^*p* < 0.001, and ^****^*p* < 0.0001 vs. LPS group).

Supplementary Figure 3 Effects of licochalcone A on mammary gland in LPS-induced mice mastitis. Mammary gland tissues from each experimental group (*n* = 10) were obtained at 24 h after LPS administration. Mammary gland tissues of **(A)** control group, **(B)** LPS group, **(C)** LPS + licochalcone A (5 mg/kg) group, **(D)** LPS + licochalcone A (10 mg/kg) group, and **(E)** LPS + licochalcone A (15 mg/kg) group.
