# Supplementary figures and images for: Licochalcone A Protects the Blood–Milk Barrier Integrity and Relieves the Inflammatory Response in LPS-Induced Mastitis
Source: Front Immunol. 2019 Feb 25;10:287. doi: 10.3389/fimmu.2019.00287 (PMC6398509; doi:10.3389/fimmu.2019.00287)

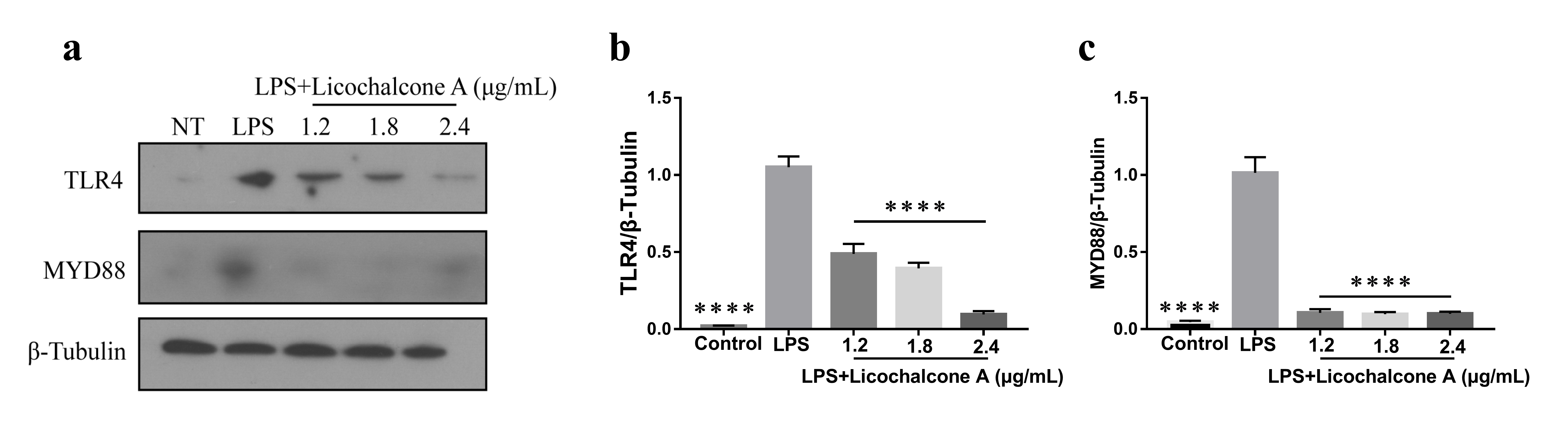

Supplement: Supplementary Figure 1 — Effects of licochalcone A on LPS-induced activation of TLR4/MYD88 signaling pathways in mMECs. (A–C) Total protein in mMECs was collected after 4 h of LPS stimulation. Licochalcone A was added 1 h before LPS stimulation. Protein levels of TLR4 and MYD88 were detected via western blot and quantitatively assessed via densitometry using β-tubulin as an internal control. Protein levels were measured using ImageJ software (http://imagej.nih.gov/ij/) and normalized to that of β-tubulin. Values are presented as means ± SD (n = 3) (*p < 0.05, **p < 0.01, ***p < 0.001, and ****p < 0.0001 vs. LPS group). [file Image_1.TIF]

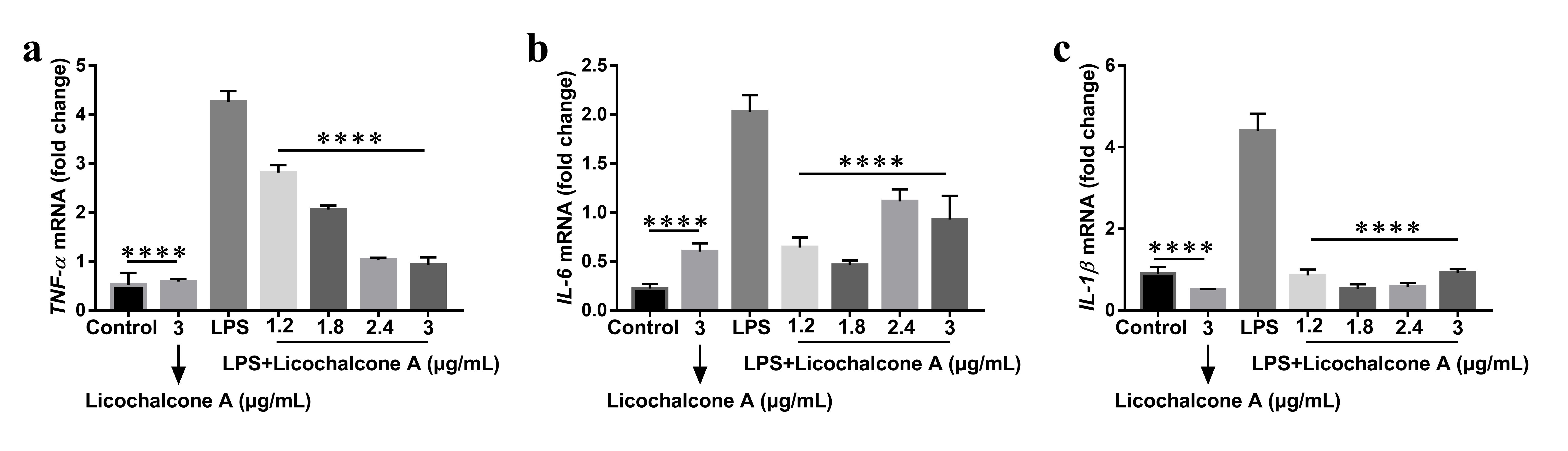

Supplement: Supplementary Figure 2 — Effects of Licochalcone A on LPS-induced inflammatory response in mouse macrophage (mMECs). The mRNA levels of IL-6 (A), IL-1β (B), and TNF-α (C), and the relative mRNA level was normalized to β-actin mRNA. Values are presented as means ± SD (n = 3) (*p < 0.05, **p < 0.01, ***p < 0.001, and ****p < 0.0001 vs. LPS group). [file Image_2.TIF]

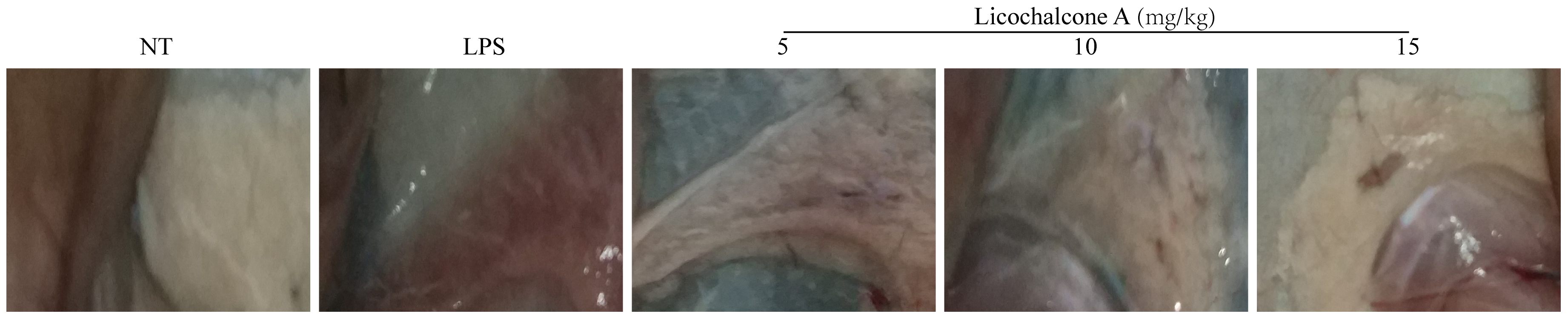

Supplement: Supplementary Figure 3 — Effects of licochalcone A on mammary gland in LPS-induced mice mastitis. Mammary gland tissues from each experimental group (n = 10) were obtained at 24 h after LPS administration. Mammary gland tissues of (A) control group, (B) LPS group, (C) LPS + licochalcone A (5 mg/kg) group, (D) LPS + licochalcone A (10 mg/kg) group, and (E) LPS + licochalcone A (15 mg/kg) group. [file Image_3.jpg]
